# Single cell transcriptomics suggest that human adipocyte progenitor cells constitute a homogeneous cell population

**DOI:** 10.1186/s13287-017-0701-4

**Published:** 2017-11-07

**Authors:** Juan R. Acosta, Simon Joost, Kasper Karlsson, Anna Ehrlund, Xidan Li, Myriam Aouadi, Maria Kasper, Peter Arner, Mikael Rydén, Jurga Laurencikiene

**Affiliations:** 1 Karolinska Institutet, Lipid Laboratory, Department of Medicine Huddinge, Novum D4, Hälsovägen 7, 14186 Stockholm, Sweden; 2 Karolinska Institutet, Department of Biosciences and Nutrition, Novum, Hälsovägen 7, 14186 Stockholm, Sweden; 3 Karolinska Institutet, Department of Medical Biochemistry and Biophysics, Scheelelaberatoriet, Scheeles väg 2, 17177 Stockholm, Sweden; 4 Karolinska Institutet, ICMC, Department of Medicine Huddinge, Novum, Hälsovägen 7, 14186 Stockholm, Sweden

**Keywords:** Single cell sequencing, Human adipose tissue, Adipocyte progenitor, Mesenchymal stem cells

## Abstract

Regulation of adipose tissue stem cells (ASCs) and adipogenesis impact the development of excess body fat-related metabolic complications. Animal studies have suggested the presence of distinct subtypes of ASCs with different differentiation properties. In addition, ASCs are becoming the biggest source of mesenchymal stem cells used in therapies, which requires deep characterization. Using unbiased single cell transcriptomics we aimed to characterize ASC populations in human subcutaneous white adipose tissue (scWAT). The transcriptomes of 574 single cells from the WAT total stroma vascular fraction (SVF) of four healthy women were analyzed by clustering and t-distributed stochastic neighbor embedding visualization. The identified cell populations were then mapped to cell types present in WAT using data from gene expression microarray profiling of flow cytometry-sorted SVF. Cells clustered into four distinct populations: three adipose tissue-resident macrophage subtypes and one large, homogeneous population of ASCs. While pseudotemporal ordering analysis indicated that the ASCs were in slightly different differentiation stages, the differences in gene expression were small and could not distinguish distinct ASC subtypes. Altogether, in healthy individuals, ASCs seem to constitute a single homogeneous cell population that cannot be subdivided by single cell transcriptomics, suggesting a common origin for human adipocytes in scWAT.

**Electronic supplementary material:**

The online version of this article (doi:10.1186/s13287-017-0701-4) contains supplementary material, which is available to authorized users.

## Introduction

White adipose tissue (WAT) dysfunction is central to the pathologies associated with overweight/obesity such as insulin resistance, type 2 diabetes, dyslipidemia, and atherosclerosis. Obesity and insulin resistance are characterized by increased fat cell size, changes in lipid/glucose metabolism, as well as increased infiltration of leukocytes, primarily macrophages [1, 2].

Adipocytes develop from adipocyte stem cells (ASCs) in a process termed adipogenesis. Given that ~ 10% of the adipocyte pool is renewed annually in adult humans, altered adipogenesis might impact on adipose tissue function [3, 4]. This notion is supported by the observation that hypertrophic WAT (few, large fat cells), in comparison to a hyperplastic phenotype (many small fat cells), is closely linked to low adipocyte turnover and a pernicious metabolic profile [3–5]. The current view is that hypertrophic obesity develops when ASC differentiation is attenuated, leading to ectopic lipid deposition in peripheral tissues such as the liver, muscle, and vessels [6]. Recent data in humans confirm that adipogenic markers in the entire ASC population correlate with fat cell size and poor metabolic measures [7]. Altogether, dysregulation of ASCs and adipogenesis appear important in the development of metabolic complications to excess body fat.

ASCs are the most abundant cell type in the stroma vascular fraction (SVF) of WAT, and murine studies have suggested the presence of several ASC populations displaying different capacities to undergo adipogenesis [8, 9]. Some markers that enrich for ASCs with marked differentiation capacity have also been identified in humans, including CD34 [10] and CD36 [11]. However, it is still unknown whether different populations of human ASCs are present in vivo.

In addition, ASCs are becoming an important source of mesenchymal stem cells (MSCs) used in allogeneic cellular therapeutics [12]. Therefore, characterization of heterogeneity of human ASCs is of great importance even for cellular therapies.

We aimed to map ASC populations in human subcutaneous WAT by single cell RNA sequencing of the total SVF of healthy individuals.

## Materials and methods

### Human subjects and adipose tissue dissociation

For single cell RNA sequencing, the SVF from subcutaneous (sc) WAT of four healthy individuals (Table 1) was isolated as described previously [13]. For flow cytometry sorting, SVF from scWAT of six healthy individuals (Table 2) was isolated and prepared as described previously [7]. The study was approved by the regional ethics board and all subjects provided their written informed consent.

**Table 1:** Characterization of patients: single cell sequencing

Patient ID	Gender	Age (years)	BMI
2014-36	Female	61	26.6
2014-37	Female	46	33.6
2014-39	Female	47	30.1
2014-124	Female	36	24.3

*BMI* body mass index

**Table 2:** Characterization of patients: fluorescence-activated cell sorting microarray

Patient ID	Gender	Age (years)	BMI
2016-76	Female	39	31.2
2016-83	Female	67	23.8
2016-86	Female	35	21.0
2016-89	Female	64	28.1
2016-96	Female	43	23.4
2016-98	Female	60	29.2

*BMI* body mass index

### Single cell capture and imaging

Loading of SVF samples on a C1 Single-Cell AutoPrep IFC microfluidic chip as well as imaging/cell selection were performed as described previously [14] and in Additional file 1: Supplemental methods.

### Amplification, tagmentation, and sequencing

RT and PCR mixes were added to the chip and samples were further processed using the C1 instrument script, which included lysis, reverse transcription, and amplification. cDNA quality was analyzed with an Agilent BioAnalyzer. All procedures including tagmentation and sequencing were as described previously [14] and in Additional file 1: Supplemental methods.

### Data analysis

Single cell RNA-sequencing data from 574 cells were analyzed in a custom Python environment. The data analysis workflow was as described in detail previously [15]. In brief, the following steps were performed: cell selection; clustering of all cells (first-level clustering); t-distributed stochastic neighbor embedding (t-SNE) visualization of all cells; identification of differential expressed genes in cell populations using negative binominal regression; clustering of ASCs (second-level clustering); rare cell detection; and pseudotemporal modeling. All procedures are described in detail in Additional file 1: Supplemental methods. The expression data were corrected for batch effects using ComBat [16] and normalized according to total molecule number before cubic spline fitting.

### Flow cytometry sorting and RNA expression profiling by microarray

Flow cytometry sorting of human WAT SVF was performed as described previously [7]. RNA was prepared from eight different cell WAT cell types (ASCs, total adipose tissue macrophages (ATMs), M1 ATMs, M2 ATMs, total T cells, CD4^+^ T cells, CD8^+^ T cells, and mature adipocytes). Ten nanograms of RNA was amplified using four cycles and loaded onto Clariom™D microarray chips. For details see Additional file 1: Supplemental methods. Microarray data have been published in GEO (https://www.ncbi.nlm.nih.gov/geo/query/acc.cgi?acc=GSE100795; token qxuxgcoojfwdpgd).

## Results

**Fig. 1:**
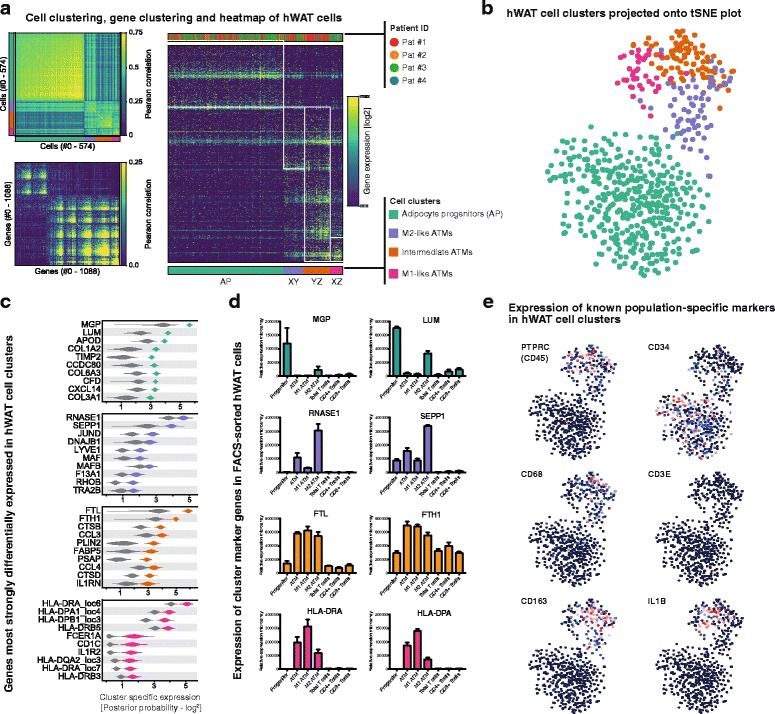
**a** First-level clustering of SVF cells from scWAT. Left: cell–cell (upper) and gene–gene (lower) distance matrices of cells and genes ordered according to cluster membership determined by first-level clustering. Pearson correlation used as distance metric. Right: heatmap showing normalized expression of genes (rows) over all cells (columns) in the dataset. Cells and genes ordered as shown on the left. Upper panel shows Patient ID membership of cells, while lower panel shows cluster membership. **b** t-SNE plot showing visualization of WAT cells in 2-dimensional space. Cells colored according to cluster membership introduced in (**a**). **c** Violin plots showing the most differentially expressed genes in each WAT cluster based on negative binominal regression analysis. A gene is defined as differentially expressed in a population if its posterior probability (PP) exceeds the PP of all other populations with at least 99% probability. Genes shown were selected from all significant differentially expressed genes according to distance between the median expression in the relevant population (colored violin) compared to second highest median expression in any other population (gray violin). **d** Expression of two top genes representing each cluster/t-SNE population (**c**) in flow cytometry-sorted WAT cell populations. Expression measurement performed by Affymetrix Clariom™D microarray, normalized values compared (*n* = 6). **e** t-SNE plots showing expression of selected WAT marker genes over the dataset. ATM adipose tissue macrophage, FACS fluorescence-activated cell sorting, t-SNE t-distributed stochastic neighbor embedding, hWAT human white adipose tissue

To identify ASC subpopulations in human scWAT, we sequenced SVF-derived single cells; 574 cells passed quality control. Subsequently, most variable genes were selected (Additional file 2: Table S1). Cell and gene clustering, as well as heatmap analysis, could separate the cells into four groups, which were present in all individuals (Fig. 1a). t-SNE visualization also suggested four major cell populations (Fig. 1b). We identified the genes that best characterized these cell groups (Fig. 1c) and examined their expression in microarrays from FACS-sorted SVF of scWAT obtained from six different patients (Fig. 1d). This showed that the largest t-SNE population represented ASCs while the remaining three populations mapped to ATMs of M1, M2, and an intermediate subtype (Fig. 1d). Analysis of the single cell transcriptome for established markers specific for ASCs and macrophages confirmed the predicted populations (Fig. 1e).

**Fig. 2:**
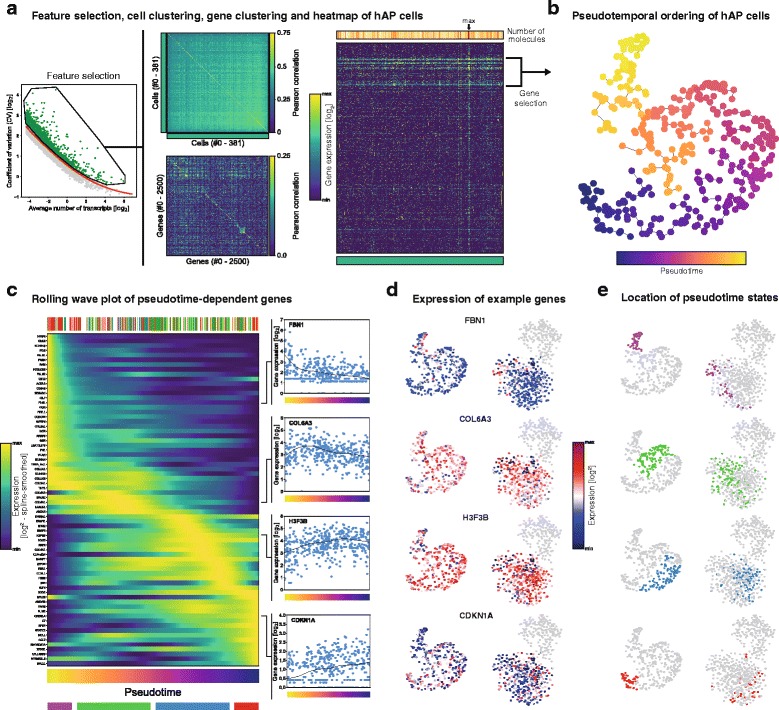
**a** Second-level clustering of ASCs. Left: cell–cell (upper) and gene–gene distance matrices (lower) of cells and genes ordered according to cluster membership determined by first-level clustering. Pearson correlation used as distance metric. Right: heatmap showing normalized expression of genes (rows) over all cells (columns) in the dataset. Cells and genes ordered as shown on the left. Upper panel shows total number of unique molecules per cell, while lower panel shows cluster membership. Apparent from both the heatmap and the cell–cell clustering, there were no apparent subpopulations of ASCs present in the data. **b** t-SNE visualization of ASCs based on gene modules selected in **a**. A minimum spanning tree through the data and the corresponding diameter path are shown. Cells colored according to position in pseudotime. **c** Rolling-wave plot showing the spline-smoothed expression patterns of significant pseudotime-dependent genes ordered according to pseudotime point of peak expression. Upper panel shows pseudotime position of ASCs colored according to patient ID. Lowest panel shows position of four pseudotime bins corresponding to the most prominent expression patterns. Example genes peaking in all four bins shown on the right. **d** Expression patterns of example genes introduced in C projected onto t-SNE visualization of ASCs (left) and SVF cells (right). **e** Position of pseudotime bins introduced in C projected onto t-SNE visualization of ASCs (left) and SVF cells (right)

Although the single cell transcriptomics revealed distinct ATM subtypes, the ASC cluster appeared homogeneous. To identify distinct ASC subtypes we selected the most variable genes within the ASC population (*n* = 381 cells, genes listed in Additional file 2: Table S2) and performed cell and gene clustering analysis summarized as a heatmap (Fig. 2a) and a t-SNE plot (Fig. 2b). However, no distinct clusters were found using these approaches. Furthermore, analysis designed to search for rare cell types [17] could also not reveal any subtypes (data not shown).

We considered that even though no specific ASC populations were detected, the cells might represent a gradient of differentiation stages. Therefore, we placed individual cells along a pseudotemporal trajectory in t-SNE space (Fig. 2b) and screened for pseudotime-dependent genes (*n* = 70 genes over significance cutoff point). Gene expression was visualized using a rolling-wave plot (Fig. 2c). Several collagen genes, *CD55*, and *Thy1* were more highly expressed in the cells localizing to the beginning of the pseudotemporal ordering, while ribosomal genes and *KLF4* were enriched toward the end. However, these genes showed only very minor differences in expression within ASCs (Fig. 2d). In conclusion, while we were able to divide ASCs into slightly different pseudotime-related states (Fig. 2e), we found no evidence of distinct ASC subtypes in human WAT.

## Discussion

To our knowledge, this is the first study reporting single cell transcriptomic data of total resident SVF cells from human WAT. Our main finding is that in healthy individuals ASCs seem to constitute a single and homogeneous population without evidence of any distinct subtypes, which is in agreement with earlier study using single cell PCR of presorted ASCs [18]. In contrast, we were able to define several ATM populations which served as an indirect quality control for our data set.

By performing pseudotemporal ordering of the cells we observed a significant gradient of gene expression for 70 genes. Previously characterized markers for committed adipocyte progenitors, such as CD34, PDGFRA, CD29, and CD36 [19], were not among these. A few other markers known to be regulated by adipogenesis or cell commitment (CD55, SFRP4, SEMA3C) [19–21] appeared to be differentially expressed along the pseudotime axis. Unfortunately, expression differences were too small and variable to enable FACS sorting of the cells belonging to the early versus late phase of pseudotemporal order.

Markers for brown (MYF5, PAX5, MYOD1 [22]) or beige/bright (MYF11 [23]) adipocyte progenitors were absent in our data set. A few cells expressed low levels of CD24 [8] while no cells expressed VSTM2A [9], suggesting that neither of these markers label ASCs in humans.

As already mentioned, several murine studies and few human studies have indicated that the ASC population is heterogeneous in the adipogenic capacity [8–11]; however, entire transcriptomes of the cells purified ex vivo have never been compared. We do not exclude that single markers might be connected with commitment to adipogenesis or osteogenesis. However, such commitment cannot be observed on the single cell transcriptome level, meaning that differences in the gene transcription level in the human ASC population are small. Clear detection of CD36 and CD34 expression as well as PDGFRA and PDGFRB expression suggests that we do not lack populations that were shown earlier to mark cells with different adipogenic capacity/commitment, but these surface markers do not correspond to specific transcriptomes of the cells.

Although our data suggest no major differences in the transcriptome of individual ASCs, we cannot exclude that differences and distinct populations may be present under specific conditions such as insulin resistance and/or detected using other approaches than those used herein (sequencing of SVF samples on the C1 Fluidigm system). In this study, we aimed to visualize heterogeneity of ASCs in healthy individuals and we cannot exclude that morbid obesity or diabetes might induce changes in the ASC population. Finally, because we only examined scWAT we cannot exclude that distinct progenitor populations may exist in visceral WAT or in brown/beige adipose tissue. Furthermore, we cannot exclude the possibility of variability occurring beyond the level of mRNA expression (e.g., epigenetics), or only being detected by gene-specific approaches. These questions were out of the scope of this report and will be addressed by future studies.

Taken together, our single cell transcriptomic approach suggests that ASCs in healthy individuals constitute a homogeneous cell population with only small variations in differentiation state.

## Additional files

Additional file 1: Supplemental methods. (DOCX 32 kb)

Additional file 2: Genes used for first-level (Supplemental table 1) and second-level (Supplemental table 2) clustering. (XLSX 132 kb)

## Abbreviations

ASC: Adipose tissue stem cell
ATM: Adipose tissue macrophage
FACS: Fluorescence-activated cell sorting
MSC: Mesenchymal stem cell
sc: Subcutaneous
SVF: Stroma vascular fraction
t-SNE: t-Distributed stochastic neighbor embedding
WAT: White adipose tissue
