# Supplementary material for: Single cell transcriptomics suggest that human adipocyte progenitor cells constitute a homogeneous cell population
Source: Stem Cell Res Ther. 2017 Nov 7;8:250. doi: 10.1186/s13287-017-0701-4 (PMC5678572; doi:10.1186/s13287-017-0701-4)
Supplement: Supplementary file 1 — Supplemental methods. (DOCX 32 kb) [file 13287_2017_701_MOESM1_ESM.docx]

**Supplemental Methods**

***Adipose tissue dissociation, single cell capture and imaging***

Adipose tissue dissociation

The stromal vascular fraction (SVF) was isolated from subcutaneous adipose tissue as described [^1^](#_ENREF_1). In brief, tissue was collagenase treated and incubated at 37° C, filtered, and washed with PBS. After several minutes the adipocytes floated, and the infranatant was collected for centrifugation at 200 x g for 10 minutes. Erythrocytes were lysed then the SVF was washed and centrifuged further. Isolated SVF was suspended in FCS supplemented with 10% DMSO and stored in liquid nitrogen.

Cell capture and imaging

Upon analysis, SVF was thawed then immediately washed with DMEM/F12 (Life Technologies) with 0.5% BSA. Cells were then filtered with CellTrics 20 μm filters (Partec GmbH, Germany) and centrifuged for 10 minutes at 200 x g. Supernatant was removed, cells were suspended in 20 μl media then counted. The cell density was adjusted to 800 cells/μl, and 1 μl RNase free-DNase [2000U/ml] (Qiagen) was added per 20 μl of cell suspension. 4 μl of C1 Suspension Reagent was added to 7 μl of cell suspension (all ‘C1’ reagents are from Fluidigm, Inc.) totaling 11μl. This 11 μl mix was then loaded on a C1 Single-Cell AutoPrep IFC microfluidic chip designed to capture cells 10-17 μm in diameter. The chip was then processed on a Fluidigm C1 instrument using the ‘mRNA Seq: Cell Load (1772x/1773x)’ script (30 minutes at 4° C). Brightfield (20x magnification) images of each capture site were acquired by a Nikon TE2000E automated microscope using μManager (<http://micro-manager.org>) [^2^](#_ENREF_2).

Quality control and selection

After each chip was loaded, images of the individual capture sites were used to determine cell diameter by an automated image analysis algorithm in MATLAB. These images were further used to manually inspect each and every capture site in order to discriminate against cells unhealthy in appearance, empty chambers, and doublet occurrence. Only chambers housing single, healthy cells were processed from this point forward.

***Amplification, tagmentation and sequencing***

Lysis, reverse transcription and PCR

RT and PCR mixes were added to the chip as previously described [^3^](#_ENREF_3). Samples were further processed using the C1 instrument script ‘mRNA Seq: RT + Amp (1772x/1773x)’, which included lysis, reverse transcription and 21 cycles of PCR. cDNA was harvested in 13 μl of C1 Harvesting Reagent; cDNA quality was analyzed with an Agilent BioAnalyzer. Yield was typically 1ng/μl.

Tagmentation and isolation of 5’ fragments

Amplified cDNA was simultaneously fragmented and barcoded by tagmentation, i.e. using Tn5 DNA transposase to transfer adaptors to the target DNA as previously described [^3^](#_ENREF_3). 100 μl Dynabeads MyOne Streptavidin C1 beads (Invitrogen) were washed in 2Å~ BWT (10 mM Tris-HCl, pH 7.5, 1 mM EDTA, 2 M NaCl, 0.02% Tween-20) then suspended in 2 ml 2Å~ BWT. Twenty microliters of beads were added to each well and incubated at room temperature for 15 min. All fractions were pooled, the beads were immobilized and the supernatant removed (thus removing all internal fragments and retaining only the 5′- and 3′-most fragments). The beads were then suspended in 100 μL TNT (20 mM Tris, pH 7.5, 50 mM NaCl, 0.02% Tween), washed in 100 μL Qiaquick PB (Qiagen), then washed twice in 100 μL TNT. The beads were then suspended in 100 μL restriction mix (1Å~ NEB CutSmart, 0.4 U/μL PvuIHF enzyme), designed to cleave 3′ fragments carrying the PvuI recognition site. The mix was incubated for 1 h at 37 °C, and then washed three times in TNT. Finally, to elute DNA, beads were suspended in 30 μL ddH2O and incubated 10 minutes at 70°C. Beads were then immediately bound to magnet and the supernatant was collected. To remove short fragments, Ampure beads (Beckman Coulter) were used at 1.8Å~ volume and eluted in 30 μL.

Read quality filtering and barcode extraction

Sequencing was performed on an Illumina HiSeq 2000. Every read that was considered valid by the Illumina HiSeq control software was processed and filtered as follows:
 a) any 3' bases with a quality score of 'B' were removed,
 b) the well-identifying barcode was extracted from the 5' end of the read,
 c) if the read ended in a poly(A)-sequence leaving less than 25 transcript-derived bases, the read was discarded,
 d) the UMI was extracted and if any of the UMI bases had a Phred score less than 17 the read was discarded,
 e) a maximum of nine template-switching generated Gs were removed from the 5' end of the transcript-derived sequence,
 f) if the remaining sequence consisted of less than six non-A bases, or a dinucleotide repeat with less than six other bases at either end, then the read was discarded.

Alignment

Reads were aligned to the genome using the Bowtie1 aligner, allowing for up to three mismatches and up to 24 alternative mappings for each read. The genome included an artificial chromosome, containing a concatamer of the ERCC control sequences. Any reads with no alignments were re-aligned against another artificial chromosome, containing all possible splice junctions arising from the exons defined by the known transcript variants. Reads mapping within these splice junctions were translated back to the corresponding actual genomic positions. The UCSC hg19 transcript models were used for the expression level calculation. If a locus had several transcript variants, the exons of these were merged to a combined model that represented all expression from the locus. To account for incomplete cap site knowledge, the 5' ends of all models were extended by 100 bases, but not beyond the 3' end of any upstream nearby exon of another gene of the same orientation.

Annotation and quantitation

The annotation step was performed barcode-by-barcode. For every unique mapping (genomic position and strand) the number of reads in each UMI was counted. Any multi-read that had one or more repeat mappings that was outside exons, was assigned randomly as one of these repeats and contributed to the summarized read count of that repeat class. Else, if it had one or more mappings to exons, it was assigned at the exon where it was closest to the transcript model 5' end, even if the sequence was repeat-like. If it had no exon mapping, it was assigned randomly at one of the mappings. After assigning reads, the number of molecules at each mapping position was determined by the number of distinct UMIs observed. To account for UMIs that stem from PCR-induced mutations or sequencing errors, any UMI represented by only a single read was excluded. The UMI count was corrected for the UMI collision probability (important only at very high UMI counts, > 2000). The expression level of each transcript model was taken as the total number of molecules at all its possible mapping positions.

***Data analysis***

Cell selection

Cells with less than 2000 unique molecules were discarded. After cell selection and quality control, 617 single cell transcriptomes remained in the dataset.

Clustering of all hWAT cells (1st level clustering)

For the 1st level clustering of all hWAT cells, a noise model was fitted as described previously [^4^](#_ENREF_4) and the 2500 genes with mean expression ≥ 0.5 molecules and the highest difference between expected and observed coefficient of variation (CV) were selected. Cells and genes were then clustered independently with the affinity propagation algorithm using Pearson correlation as distance metric. Cluster numbers were selected based on biological considerations and Bayesian Information Criterion (BIC) score. In order to allow a more precise clustering of cells, all gene modules showing a distinct expression pattern (as opposed to plain noise) were selected after the first round of clustering and the cell clustering was subsequently repeated with this more restricted gene set. The 1st level clustering yielded four robust cell clusters based on the expression of seven selected gene modules.

tSNE visualization of all hWAT cells

Non-linear dimensionality reduction of the full dataset was performed with t-stochastic neighbor embedding (tSNE). Pearson correlation between cells – considering the same set of genes as in the 1st level clustering – was used as distance metric. The following parameters were used: perplexity = 25.0, early_exaggeration = 2.0, learning_rate = 1200.0.

Identification of differential expressed genes in hWAT populations using negative binominal regression

Differentially expressed genes were identified using a Bayesian negative binominal regression model as described previously [^4^](#_ENREF_4). A gene was considered differentially expressed in a cell population if its posterior probability (PP) exceeded the PP of all other groups with more than 99% probability.

Clustering of hAPs (2nd level clustering)

For clustering of hAPs, all cells from the 1st level hAP cluster (397 cells) were selected and feature selection was performed as described above. As shown in Figure 2A, 2nd level clustering did not yield any distinctly differentially regulated gene modules or robust clusters of cells.

tSNE visualization and pseudotemporal ordering of hAPs

In order to analyze the hAP data for small or gradual differences in gene expression, all gene modules which showed a strong expression pattern in hAPs (compare Figure 2A) were selected. Based on this gene set, tSNE was performed as described above using the following parameters: perplexity = 20.0, early_exaggeration = 2.0, learning_rate = 30.0. Based on their position in the resulting tSNE plot, which shows a clear degree of graduality, AP cells were brought into a distinct order (pseudotime) as described previously [^4^](#_ENREF_4)^,^[^5^](#_ENREF_5). In brief, a minimum spanning tree (MST) was generated from all hAP cells based on their distances in tSNE space and the longest path through the MST (diameter path) was determined. The cells were subsequently brought into an order which minimizes the total distance while remaining within the constraints of the diameter path.

Identification of pseudotime-dependent genes

In order to model gene expression along pseudotime, a cubic spline model with 5 degrees of freedom was fitted for each gene with mean expression ≥ 0.1 molecules in hAPs. The cubic spline-fitted model was then compared to a restricted model (no change along pseudotime) using the approximate likelihood test. All genes with Benjamini-Hochberg corrected p-values ≤ 0.01 were considered pseudotime-dependent.

In order to make sure that the detected differences in gene expression along pseudotime are not just an artifact due to batch / patient effects or differences in total mRNA yield between cells, the expression data was corrected for batch effects using ComBat [^6^](#_ENREF_6) and normalized according to total molecule number before cubic spline fitting. In total, we detected 70 pseudotime-dependent genes.

Rare cell detection

To ensure that no population(s) of rare but transcriptionally distinct cells are hidden among the hAPs, we used a modification of the approach introduced previously [^7^](#_ENREF_7). In brief, we fitted a noise model to model the relationship between number of molecules and variance. For each gene with mean expression ≥ 0.05 molecules, we then used this model to determine the parameters for its expected negative binominal distribution. This allowed to us determine the probability that a certain gene is expressed in line with expectations in a certain cell and subsequently enabled us to get a measure of overall deviation from the expected gene expression distributions for each cell (sum of –log10 P-values over all genes in a cell).

We considered all AP cells over the 90th percentile of distinctiveness as potential rare cells. However, subsequent clustering of the 18 so-defined potential rare cells showed that they do not share any differentially expressed gene signatures. Therefore, we concluded that these cells most likely represent particularly noisy cells without them constituting one or more rare cell populations.

***Flow cytometry sorting and RNA expression profiling by microarray***

Flow cytometry sorting of hWAT SVF pools from 3 lean donors (22.7 ± 1.5 kg/m^2^, 48.3 ± 16.7 years) and 3 overweight/obese donors (29.5 ± 1.6 kg/m^2^, 54.3 ± 13.4 years) was performed as previously described [^8^](#_ENREF_8). Adipocyte progenitors, total adipose tissue macrophages, M1 macrophages, M2 macrophages, total T-cells, CD4+ T-cells, CD8+ T-cells were collected in duplicate. In addition, mature adipocytes were collected.

Ten ng of RNA per fraction was amplified using 4 cycles and loaded on Clariom™D human microarray chips in accordance with manufacturer’s recommendations (Affymetrix). Data were analyzed using the SST-RMA algorithm in Expression Console (Affymetrix). Data have been submitted to NCBI GEO database (reviewer link: https://www.ncbi.nlm.nih.gov/geo/query/acc.cgi?acc=GSE100795)

Further information

More detailed information regarding the described analyses can be found in the Supplemental Experimental Procedures of [^4^](#_ENREF_4). Furthermore, the corresponding Python scripts and Jupyter notebooks containing the complete analysis workflow are shared on <https://github.com/kasperlab>.

**References**

**1.** van Harmelen V, Skurk T, Hauner H. Primary Culture and Differentiation of Human Adipocyte Precursor Cells. In: Picot J, ed. *Human Cell Culture Protocols*. Totowa, NJ: Humana Press; 2005:125-135.

**2.** Stuurman N, Edelstein AD, Amodaj N, Hoover KH, Vale RD. Computer Control of Microscopes using μManager. *Current protocols in molecular biology / edited by Frederick M. Ausubel ... [et al.].* 2010;CHAPTER:Unit14.20-Unit14.20.

**3.** Islam S, Zeisel A, Joost S, et al. Quantitative single-cell RNA-seq with unique molecular identifiers. *Nat Meth.* 2014;11(2):163-166.

**4.** Joost S, Zeisel A, Jacob T, et al. Single-Cell Transcriptomics Reveals that Differentiation and Spatial Signatures Shape Epidermal and Hair Follicle Heterogeneity. *Cell Systems.* 2016;3(3):221-237.e229.

**5.** Trapnell C, Cacchiarelli D, Grimsby J, et al. The dynamics and regulators of cell fate decisions are revealed by pseudotemporal ordering of single cells. *Nat Biotech.* 2014;32(4):381-386.

**6.** Johnson WE, Li C, Rabinovic A. Adjusting batch effects in microarray expression data using empirical Bayes methods. *Biostatistics.* 2007;8(1):118-127.

**7.** Grun D, Lyubimova A, Kester L, et al. Single-cell messenger RNA sequencing reveals rare intestinal cell types. *Nature.* 2015;525(7568):251-255.

**8.** Acosta JR, Douagi I, Andersson DP, et al. Increased fat cell size: a major phenotype of subcutaneous white adipose tissue in non-obese individuals with type 2 diabetes. *Diabetologia.* 2016;59(3):560-570.
